# Randomized clinical trial on safety of the natriuretic peptide ularitide as treatment of refractory cirrhotic ascites

**DOI:** 10.1097/HC9.0000000000000481

**Published:** 2024-06-27

**Authors:** Rasmus H. Gantzel, Emilie E. Møller, Niels K. Aagaard, Hugh Watson, Peter Jepsen, Henning Grønbæk

**Affiliations:** 1 Department of Hepatology and Gastroenterology, Aarhus University Hospital, Aarhus N, Denmark; 2 Department of Clinical Medicine, Aarhus University, Aarhus, Denmark; 3 Department of Medicine, Regional Hospital Gødstrup, Herning, Denmark; 4 Medical Development and Translational Sciences, Evotec ID, Lyon, France

## Abstract

**Background:**

Sodium and water retention is a mainstay of the pathophysiology leading to ascites formation in patients with advanced cirrhosis. Refractory ascites denotes the most severe ascites status with limited treatment options and a poor prognosis. We investigated the efficacy and safety of the natriuretic peptide ularitide in patients with refractory cirrhotic ascites.

**Methods:**

We conducted a randomized placebo-controlled trial investigating ularitide to manage refractory ascites. Until trial termination after interim analyses, we randomized 17 participants in a 2:1 ratio between ularitide (n=11) and placebo (n=6). While hospitalized, the participants received treatment for up to 48 hours. The primary efficacy endpoint was a change in renal water excretion, and secondary end points included changes in renal sodium excretion rate and body weight. The starting dose was 30 ng/kg/min, though later reduced to 20 for safety reasons.

**Results:**

In contrast to the study hypothesis, the mean urine production decreased after 24 hours of ularitide treatment compared with the baseline level (22.8 vs. 47.5 mL/h, *p*=0.04) and decreased more in participants randomized to ularitide than placebo (24.7 vs. −6.2 mL/h, *p*=0.05). Ularitide did not increase the renal sodium excretion rate or reduce the weight gain. The incidence rate ratio of adverse reactions in ularitide versus placebo was 8.5 (95% CI: 2–35, *p*=0.003). Participants treated with ularitide developed serious blood pressure reductions, impacting their renal responsiveness.

**Conclusions:**

Ularitide in doses of 20–30 ng/kg/min did not benefit urine production and renal sodium excretion rate in patients with refractory ascites. The participants randomized to ularitide overall developed more adverse reactions than placebo. EudraCT no. 2019-002268-28.

## INTRODUCTION

Ascites formation is the most frequent sign of decompensation in patients with cirrhosis and portal hypertension.^1^ More than half of the patients develop ascites within 10 years after their diagnosis of cirrhosis.^2^ One-tenth of these patients progress to refractory ascites, where dietary and medical treatments are ineffective or induce intolerable side effects and face poor survival probabilities.^3–6^ New pharmacotherapies are warranted to reduce the burden of ascites and the need for large-volume paracentesis in the subset of patients who are ineligible for TIPS insertion, implantation of an automated low-fluid ascites pump (Alfapump), or liver transplantation.^2,3,7^

Natriuretic peptides are essential for the maintenance of cardiovascular and renal homeostasis.^8^ Especially the renal paracrine natriuretic peptide urodilatin may counterbalance sodium retention in patients with cirrhotic ascites.^9^ Urodilatin stimulates guanylate cyclase-coupled natriuretic peptide receptors in the collecting ducts, leading to suppression of renal water and sodium reabsorption, thereby increasing diuresis and natriuresis.^10^ The results from 2 pilot clinical trials indicate that patients with cirrhotic ascites can increase their renal sodium and water excretion after 60–90 minutes of continuous i.v. infusion of ularitide, a synthetic equivalent to urodilatin.^11,12^ Both pilot studies applied a dose of 20 ng/kg/min, and overall, the patients with the most advanced cirrhosis and avid sodium retention had the lowest renal effects.^12,13^

The current randomized placebo-controlled clinical trial was designed to prove the concept that up to 48 hours of continuous ularitide infusion is safe and effective in inducing and maintaining clinically meaningful renal sodium and water excretion in patients with refractory cirrhotic ascites compared with placebo treatment. Based on results from an interim analysis, the trial was prematurely terminated.

## METHODS

### Participants

The methods are described in the published trial protocol.^14^ To recap, the trial was conducted as a single-center, double-blinded study at the Department of Hepatology and Gastroenterology, Aarhus University Hospital, Aarhus, Denmark. Patients were recruited from August 2020 to November 2022. Written informed consent to participate was obtained from all participants. All included patients were randomized to continuous i.v. infusion with ularitide or placebo in a 2:1 ratio and the treatment was administered only during hospitalization. An unequal randomization sequence was chosen to mitigate the likelihood of withdrawals and to prioritize the collection of safety data for ularitide.^14^

Study inclusion was restricted to patients with cirrhosis and refractory ascites,^3^ age ≥18 years, urine (U)-sodium excretion rate <60 mmol/d, plasma (P)-creatinine <150 µmol/L, Child-Pugh score of 7–13, total P-bilirubin <150 µmol/L, and resting systolic blood pressure >95 mm Hg. The exclusion criteria included an episode of spontaneous bacterial peritonitis or gastrointestinal bleeding within the past 2 weeks, proteinuria >500 mg/d, blood (B)-hemoglobin <5.5 mmol/L, primary kidney disease, congestive heart failure, acute-on-chronic liver failure, and ongoing HE of West-Haven grade 2–4.

The primary efficacy endpoint was the change in urine volume at 24 hours postinfusion start versus baseline. The main secondary end points included the change in U-sodium excretion rate at 24 hours postinfusion start versus baseline and the change in body weight at the end of treatment versus baseline.^14^ Treatment responsiveness was defined as (1) urine volume increase of ≥100% at 2 hours post-treatment start versus baseline, (2) urine volume increase of ≥ 50% at 24 hours post-treatment start versus baseline, (3) increase in urine sodium excretion rate by 100% at the end of treatment (24–48 hours) vs. baseline, or (4) body weight reduction by ≥2 kg at the end of treatment (24–48 hours) versus baseline. Also, the changes in efficacy end points were compared between the 2 treatment groups.

### Dose selection and protocol amendments

In the 2 previous ularitide pilot studies, the diuretic response to 20 ng/kg/min was lowest in patients with refractory ascites compared to nonrefractory ascites.^11,12^ Moreover, the patients with refractory ascites showed varying responses, suggesting that the dose of 20 ng/kg/min may have been too low to induce a clinically significant renal response for this heterogenous group of patients with advanced cirrhosis. Therefore, we designed a treatment protocol with a higher starting dose but with the possibility of individualized dose titration.^14^ The first 8 participants were treated following the original trial protocol, dictating a starting dose of 30 ng/kg/min with a dose increase to 45 ng/kg/min if the urine production failed to double after 2 hours of treatment compared with baseline. The protocol also gave the possibility to reduce the dose to 15 ng/kg/min in case the systolic blood pressure dropped below 90 mm Hg.^14^ However, due to a high frequency of hypotensive episodes judged to influence the renal efficacy measures, an amendment to the protocol was implemented in September 2021. Thus, the last 9 participants were treated with a dosing regimen reduced by one-third compared with the original regimen, ie, a starting dose of 20 ng/kg/min, a possible dose increase to 30 ng/kg/min, and a safety dose reduction to 10 ng/kg/min.

### Sample size calculation, interim analysis, and statistical approaches

The sample size of 25 ularitide treatments and 13 placebo treatments was estimated using data from the largest ularitide pilot study, assuming an increased urine production of 2 mL/min for ularitide treatment and −1 mL/min for placebo.^11,14^ This sample size achieves 80% power to reject the hypothesis of equal means when applying a significance level of 5%.^14^ However, we continued to observe a high rate of adverse reactions regardless of the protocol amendment introduced halfway through the trial. Thus, after completing 17 participants, an independent external biostatistician performed a safety interim analysis. The trial investigators remained blinded to the treatments. The changes in (1) urine volume and (2) U-sodium excretion rate at 24 hours postinfusion start versus baseline, and (3) body weight at the end of treatment versus baseline were compared between ularitide and placebo using one-sided unpaired parametric tests. Paired statistical tests were used to investigate changes within each treatment arm. The incidence rates of adverse reactions in the ularitide and placebo groups were compared using a Poisson regression analysis. The incidence of stopping criteria leading to a dose reduction between the treatments was tested with Fisher exact test.

After the safety interim analyses,^15^ data examinations included the subsequent prespecified secondary efficacy end points to estimate the number of treatment responders in each treatment arm and to investigate the course of renal, neuro-hormonal, pharmacokinetic, and clinical measures during the treatment period. Furthermore, explorative subgroup analyses were performed to investigate if different treatment doses, baseline creatinine levels, spironolactone therapy, baseline systolic blood pressure, and baseline concentration of P-renin and P-aldosterone impacted efficacy end points. Changes within each treatment arm and between-group differences were investigated with the Kruskal-Wallis rank-based test, the Wilcoxon rank-sum test, the unpaired *t* test, and Fisher exact test as appropriate. Correlations were investigated using the Spearman rank-based test. Restricted cubic splines were used to assess the dynamics in resting systolic blood pressure.

### Trial registration

The trial was registered at ClinicalTrials.gov (NCT04311489) and the EudraCT register (2019-002268-28) with approvals from the Danish Medicines Agency and the Regional Ethics Committee (no. 1-10-72-177-19). The trial was conducted in accordance with the Declarations of Helsinki and Istanbul and adhered to the principles for Good Clinical Practice.

## RESULTS

### Background characteristics

**FIGURE 1:**
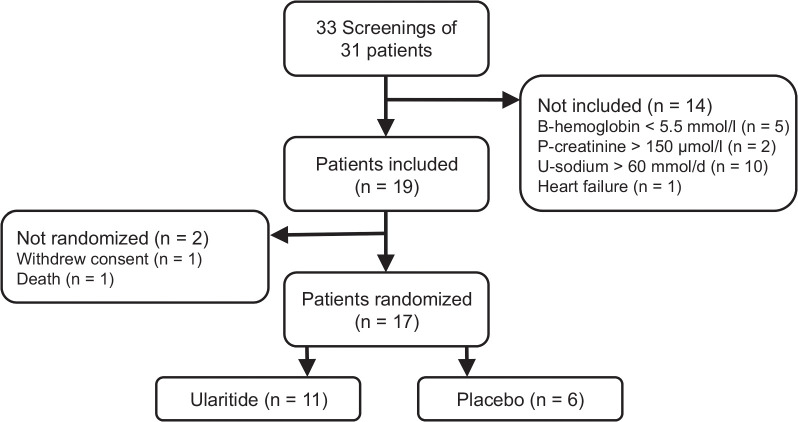
Flowchart of patient recruitment. Abbreviations: B, blood; P, plasma; U, urine; d, day.

Thirty-one patients provided written consent to participate in the trial. Of these, 17 patients were included and randomized after screening for inclusion and exclusion criteria (Figure 1). The baseline characteristics were similar between the 11 participants randomized to ularitide treatment compared with the 6 participants randomized to the placebo arm (Table 1).

**TABLE 1:** Baseline characteristics

	Summary statistics			
	Randomized (n=17)			
Variables	Ularitide (n=11)	Placebo (n=6)	All (n=17)	Excluded/withdrawn (n=14)
Demographics				
Age (y), median (IQR)	70 (60–77)	55 (53–68)	65 (53–71)	56 (54–74)
Male sex, n (%)	7 (64)	4 (67)	11 (65)	12 (86)
Anthropometrics, median (IQR)				
Height (cm)	174 (163–178)	174 (170–176)	174 (169–177)	176 (170–180)
Body weight (kg)	78 (63–85)	73 (68–83)	73 (65–84)	82 (78–102)
Body mass index	24.7 (20.8–29.4)	23.1 (22.0–28.7)	24.6 (22.0–28.7)	27.8 (19.1–31.1)
Cirrhosis etiologies, n (%)				
Alcohol	6 (55)	4 (67)	10 (59)	11 (79)
MAFLD	1 (9)	0	1 (6)	1 (7)
Alcohol+MAFLD	1 (9)	1 (17)	2 (12)	0
PSC	1 (9)	0	1 (6)	0
Toxic hepatitis	1 (9)	0	1 (6)	1 (7)
Budd-Chiari syndrome	0	1 (17)	1 (6)	0
Cryptogenic	1 (9)	0	1 (6)	1 (7)
Time since cirrhosis diagnosis (mo), median (IQR)	25 (9–47)	24 (9–65)	25 (9–47)	52 (23–104)
Time since ascites diagnosis (mo), median (IQR)	20 (4–47)	27 (9–65)	20 (5–47)	31 (12–66)
Refractory ascites debut (mo), median (IQR)	5 (2–6)	7 (6–12)	5 (3–6)	4 (1–6)
Refractory ascites characteristics, n (%)				
Diuretic-intractable	8 (73)	4 (67)	12 (71)	10 (71)
Diuretic-resistant	3 (27)	2 (33)	5 (29)	4 (29)
Diuretic therapy, median (IQR)				
Daily spironolactone (mg)	50 (0–100)	0 (0–100)	0 (0–100)	25 (0–200)
Daily furosemide (mg)	80 (20–120)	20 (0–80)	60 (0–120)	120 (80–160)
No diuretic treatment, n (%)	2 (18)	2 (33)	4 (24)	2 (14)
Paracentesis characteristics, median (IQR)				
Paracentesis past 3 mo	4 (3–7)	5 (3–8)	4 (3–7)	4 (3–6)
Ascites removed per drainage (l)	7.4 (6.0–10.4)	9.2 (5.0–11.9)	8.1 (6.0–10.4)	8.5 (6.5–13.1)
Circulation at screening, median (IQR)				
Systolic blood pressure (mm Hg)	116 (106–135)	111 (102–123)	116 (106–131)	122 (108–133)
Diastolic blood pressure (mm Hg)	66 (54–79)	69 (62–78)	67 (56–78)	68 (65–79)
Heart rate (beats/min)	85 (66–93)	82 (76–94)	85 (68–93)	81 (76–95)
Blood biochemistry, median (IQR)				
P-albumin (g/L)	30 (29–30)	27 (22–30)	30 (28–30)	30 (29–33)
P-bilirubin (µmol/L)	23 (16–60)	22 (14–33)	23 (16–45)	19 (12–22)
P-ALT (U/L)	24 (18–62)	30 (14–45)	24 (18–45)	29 (20–40)
P-alkaline phosphatase (U/L)	154 (102–256)	135 (115–220)	141 (115–220)	176 (95–218)
INR	1.3 (1.3–1.5)	1.3 (1.2–1.6)	1.3 (1.3–1.5)	1.3 (1.2–2.0)
P-sodium (mmol/L)	133 (129–135)	130 (123–132)	132 (127–135)	133 (129–140)
P-potassium (mmol/L)	4.2 (3.9–4.4)	4.6 (3.8–5.2)	4.2 (3.9–4.4)	3.9 (3.8–4.4)
P-creatinine (µmol/L)	103 (77–118)	100 (61–117)	103 (77–117)	106 (87–125)
B-platelets (×10^9^)	97 (85–186)	227 (74–326)	108 (85–195)	152 (86–198)
B-hemoglobin (mmol/L)	6.9 (6.2–8.1)	6.8 (6.4–7.0)	6.9 (6.2–7.5)	6.2 (5.3–7.6)
Child-Pugh score	8 (8–10)	9 (8–10)	8 (8–10)	8 (8–9)
MELD-Na	21 (14–26)	23 (21–29)	22 (18–26)	20 (12–30)
MELD 3.0	18 (14–21)	21 (17–22)	19 (14–22)	15 (11–24)
P-renin (×10^−3^ IU/l)	>550 (183–>550)	>550 (240–>550)	>550 (240–>550)	NA
P-aldosterone (pmol/L)	3432 (2388–9777)	8532 (3533–9781)	4925 (3019–9777)	NA
P-copeptin (pmol/L)	27.1 (14.1–35.6)	24.4 (19.6–52.5)	27.1 (19.6–35.6)	NA
Urine biochemistry, median (IQR)				
24-h volume (mL)	1000 (630–1360)	975 (700–1530)	1000 (700–1360)	1725 (900–2500)
U-sodium (mmol/L)	16 (10–27)	13 (9–17)	16 (<10–19)	53 (19–75)
U-sodium excretion rate (mmol/d)	24 (<10–37)	11 (<10–35)	19 (<10–37)	85 (13–75)
U-potassium (mmol/L)	32 (23–46)	32 (24–41)	32 (23–44)	32 (23–36)
U-potassium excretion rate (mmol/d)	32 (26–44)	39 (12–57)	32 (26–46)	41 (29–68)
U-creatinine (mmol/L)	6.7 (4.4–9.2)	7.5 (6.4–10.3)	6.9 (5.2–9.2)	4.4 (3.5–5.5)
U-creatinine excretion rate (mmol/d)	7.1 (4.6–8.3)	7.0 (6.2–7.7)	7.1 (5.9–8.2)	7.2 (6.1–9.1)
GFR-24h-creatinine-clearance (mL/min)	40 (27–69)	49 (39–75)	45 (35–69)	46 (33–63)
U-albumin (mg/day)	3 (2–7)	3 (2–4)	3 (2–6)	5 (2–29)

*Note:* Background and baseline characteristics of all patients screened for the trial inclusion.

Abbreviations: ALT, alanine aminotransferase; B, Blood; INR, international normalized ratio; MELD, Model for End-Stage Liver Disease; MELD-Na, MELD sodium; GFR, glomerular filtration rate; NA, not applicable; MAFLD, metabolic-associated fatty liver disease; P, plasma; PSC, primary sclerosing cholangitis; U, urine.

### Results of the interim analysis

**FIGURE 2:**
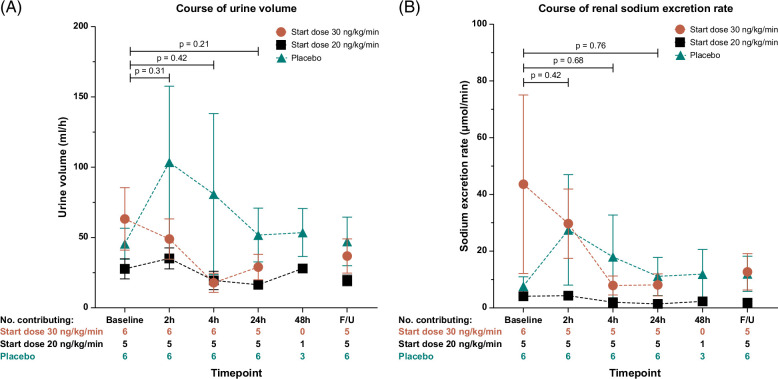
The absolute changes in (A) urine volume and (B) renal sodium excretion rate at the scheduled time points. The treatment arm is divided into the 2 dose regimens used with starting doses 30 ng/kg/min (red) or 20 ng/kg/min (black). Results are given as mean±SEM. Abbreviations: h, hours; F/U, post-treatment follow-up.

For the participants randomized to ularitide, the mean urine production reduced by 24.7±12.2 mL/h (mean±SEM), and the mean U-sodium excretion rate reduced by 21.8±17.8 µmol/min (mean±SEM) after 24 hours of treatment compared with the baseline. The mean body weight increased by 0.98 kg at the end of treatment compared with the baseline. Also, the ularitide-treated participants had lower mean urine production and mean U-sodium excretion rate after 24 hours of treatment compared with the baseline than observed for the participants treated with placebo (between-group differences of 30.9±17.5 mL/h and 25.1±18.2 µmol/min, respectively; Figure 2A, B and Supplemental Figure S1, http://links.lww.com/HC9/A943).

The incidence rate of adverse reactions was 2.8 per participant treated with ularitide and 0.3 per participant treated with placebo. Consequently, the incidence rate ratio was 8.5 (95% CI: 2.0–35, *p*=0.003). The incidence of resting systolic blood pressure drops below 90 mm Hg, leading to a dose reduction, was higher in ularitide-treated than placebo-treated participants (*p*=0.043).

These results from the interim analyses forced the trial termination and unblinding to permit investigations of the remaining secondary efficacy end points, as well as explorative subgroup analysis on the 2 dose regimens used. The proportion of treatment responders was 50% (3 of 6) among participants randomized to placebo and 27% (3 of 11) among participants randomized to ularitide.

### Urine volume

**FIGURE 3:**
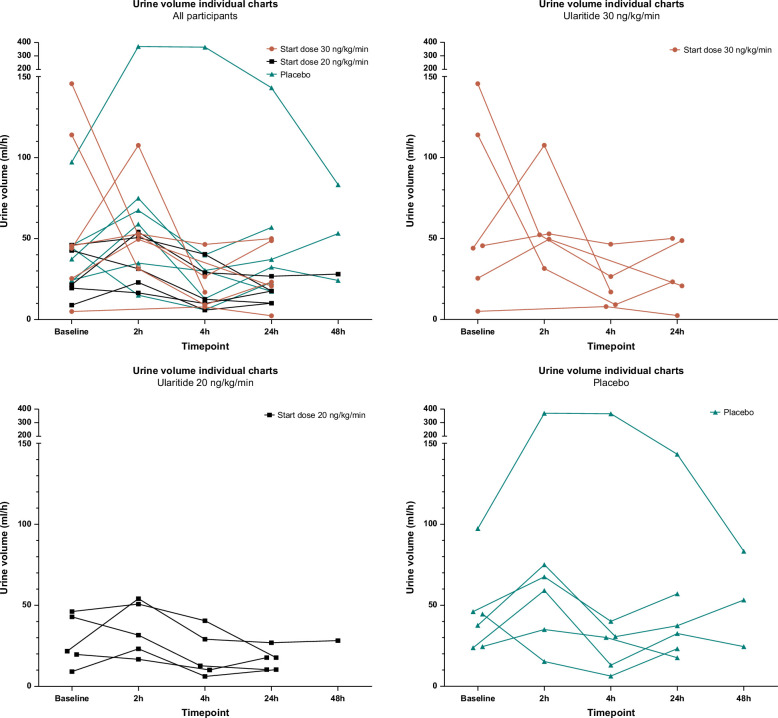
Individual-level spaghetti plots of the dynamics in urine volume for patients randomized to ularitide 30 ng/kg/min (red), ularitide 20 ng/kg/min (black), and placebo (green). Abbreviation: h, hours.

No participants in the ularitide treatment arm increased their urine production during the treatment course from baseline to 24 hours postinfusion start. One participant in the placebo arm had a marked diuretic response that impacted the overall renal effects of the placebo treatment. However, after 24 and 48 hours of treatment, the overall urine production from the placebo treatment returned to the baseline level (Figure 2A and Figure 3).

### Renal sodium excretion rate

The response pattern for renal sodium excretion rate was similar to that observed for the urine volume, with a negative renal effect from ularitide treatment and no overall effect from placebo treatment (Figure 2B).

### Body weight and waist circumference

The changes in body weight, standing waist circumference, and supine waist circumference measures were similar for ularitide and placebo treatment, showing slight increases (Figure 4A–C).

FIGURE 4The absolute changes in (A) body weight, (B) waist circumference in standing position, (C) waist circumference in supine position, (D) the renal solute-free water clearance, (E) P-creatinine, (F) P-aldosterone, (G) P-copeptin, and (H) P-cGMP during the treatment course. For body weight and waist circumference, the “24 h/end” reflects for the majority of participants the measure at the end after 24 hours treatment, while it reflects for a few participants the measure performed when stopped before 24 hours or when completing 48 hours of infusion. Results are given as mean±SEM. Abbreviations: h, hours; F/U, post-treatment follow-up; P, plasma; cGMP, cyclic guanosine monophosphate.

**Figure:**
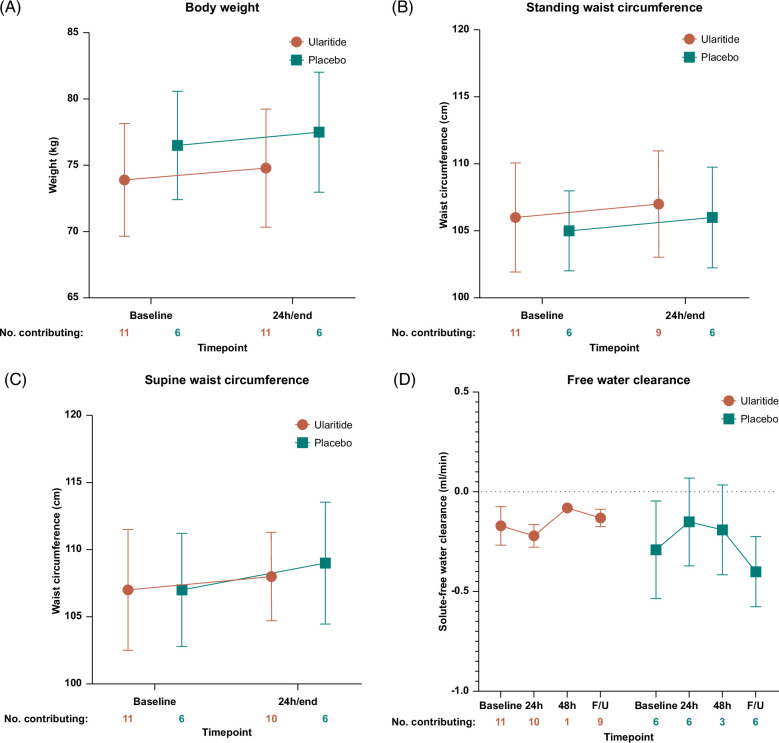

**Figure:**
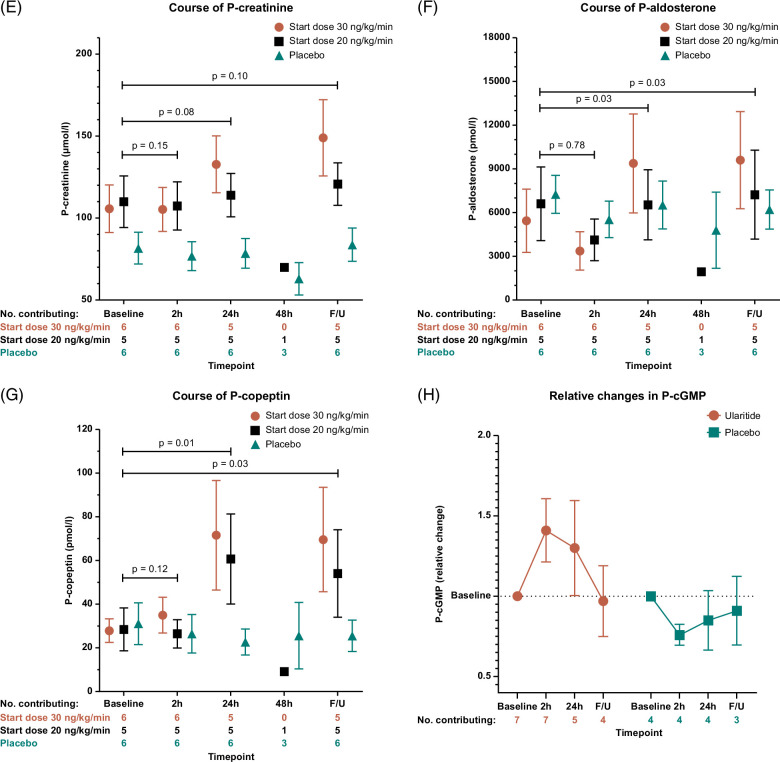

### Renal function

P-creatinine was not influenced by placebo treatment. Compared with the baseline, P-creatinine levels in the ularitide-treated participants increased after 24 hours of treatment and 3–6 hours post-treatment recovery (Figure 4E). Participants starting with the ularitide dose of 20 ng/kg/min had a smaller increase in P-creatinine than participants starting at 30 ng/kg/min. Neither ularitide nor placebo increased renal solute-free water clearance, which remained negative throughout the treatment period, reflecting water retention (Figure 4D). For the participants randomized to ularitide, we found no statistically significant correlation between the absolute increase in P-creatinine and the reduction in urine volume from the baseline to 24 hours of treatment (rho=0.45, *p*=0.19; Supplemental Figure S2, http://links.lww.com/HC9/A944).

### Hormone concentrations

Compared with the baseline, the P-aldosterone concentrations decreased after 2 hours of treatment, irrespective of treatment arm or dose (Figure 4F). The P-aldosterone concentration returned to baseline after 24 hours of placebo and 20 ng/kg/min ularitide treatments and increased above the baseline level with the ularitide dose of 30 ng/kg/min (Figure 4F). This increase persisted at the 3–6 hours postinfusion follow-up. Considering only the participants randomized to ularitide, we observed no statistically significant correlations between the increase in urine volume and the reduction in P-aldosterone concentration from the baseline to 2 hours of treatment (rho=0.13, *p*=0.71) or the increase in P-aldosterone concentration from the baseline to 24 hours of treatment (rho=0.21, *p*=0.56; Supplemental Figure S2, http://links.lww.com/HC9/A944).

The baseline P-renin concentration was >550 ×10^−3^ IU/L for 11 participants, and thus above the limit of quantification. Considering the 4 ularitide-treated participants with quantifiable P-renin concentrations, the dynamics were inconsistent during the treatment.

Copeptin constitutes a precursor fragment of vasopressin and thereby a surrogate marker of the vasopressin level. Placebo did not influence the P-copeptin concentration. In contrast, participants receiving ularitide increased P-copeptin after 24 hours of treatment compared with the baseline level (Figure 4G).

The natriuretic peptide receptor’s second messenger, cyclic GMP, was measured in plasma to document the intended stimulatory effects of the study drug. The P-cyclic GMP concentration increased after 2 and 24 hours of treatment for patients exposed to ularitide (Figure 4H).

### Safety

**FIGURE 5:**
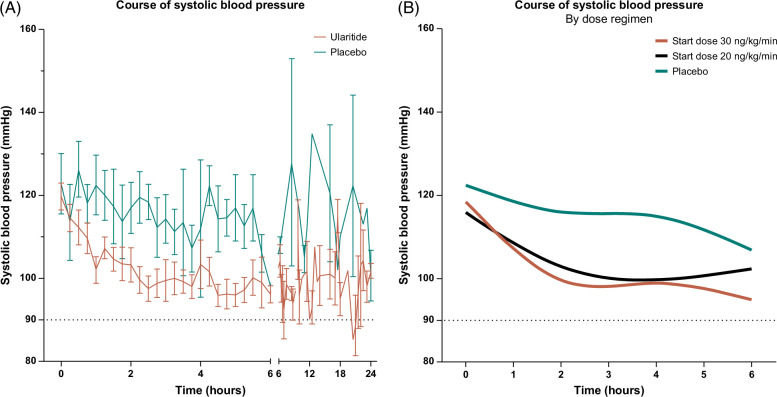
The course of systolic blood pressure from (A) baseline until end of treatment for ularitide and placebo and (B) baseline to six hours post-treatment start for ularitide dose 30 ng/kg/min (red), ularitide dose 20 ng/kg/min (black), and placebo (green). Results are given as mean±SEM and cubic spline curves using four knots.

The resting arterial blood pressure was intensively monitored during the first 6 hours of treatment. Participants randomized to ularitide and placebo had similar mean baseline systolic blood pressure (120 vs. 123 mm Hg, *p*=0.66) and heart rate (75 vs. 84 beats/min, *p*=0.20). A rapid and persistent decrease in systolic blood pressure was observed for ularitide-treated participants, while participants in the placebo arm only experienced a minor decrease (Figure 5A). The course of systolic blood pressure during the initial 6 hours of treatment was largely unaffected by the dose regimen used, ie, 30 versus 20 ng/kg/min. However, the decline was less dramatic, with earlier plateauing for 20 ng/kg/min (Figure 5B). We found no statistically significant correlations between the absolute reduction in urine volume and the reductions in mean systolic blood pressure from the baseline (Supplemental Figure S3, http://links.lww.com/HC9/A945).

The incidence of stopping criteria leading to a dose reduction, exclusively hypotensive episodes, was similar between the dose regimen of 30 ng/kg/min (4 of 6=67%) and 20 ng/kg/min (2 of 5=40%, *p*=0.57).

**FIGURE 6:**
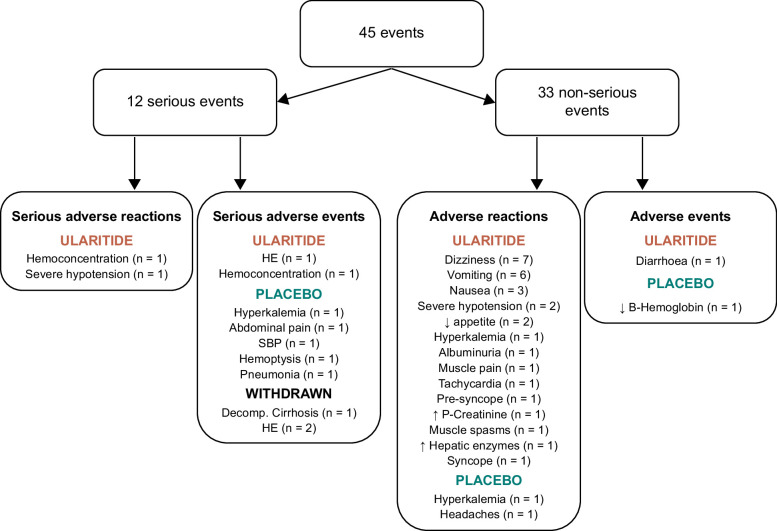
Flowchart of adverse events registered during the trial period and the distribution between the ularitide (red) and placebo (green) arms. Abbreviation: SBP, spontaneous bacterial peritonitis.

A total of 45 adverse events were registered during the trial period among the 19 included patients. Twelve serious events were recorded, of which 2 were judged as related to the study drug and categorized as serious adverse reactions. When considering the 33 adverse reactions judged as related to the study drug, 31, including the two serious ones, were registered in participants randomized to ularitide treatment (Figure 6). These numbers formed the basis for the statistics on safety end points performed in the interim analysis. The 31 adverse reactions recorded in the ularitide arm were distributed equally between the 2 treatment dose regimens (*p*=1.00). All ularitide-treated participants presented their first adverse reaction between 2 and 47 hours after the start of the infusion, without difference between the 30 ng/kg/min (8.2±4 hours; mean±SEM) and 20 ng/kg/min (6.8±4 hours; mean±SEM). There was no difference in the severity on a scale from 1 to 3 of the adverse reactions between the 2 dose regimens (*p*=0.17).

## DISCUSSION

We present the results from a randomized placebo-controlled clinical trial investigating the natriuretic peptide ularitide as a potential treatment of refractory ascites in patients with cirrhosis. Ularitide doses of 30 and 20 ng/kg/min did not increase the urine volume or sodium excretion rate after 24 hours of continuous i.v. infusion. The participants randomized to ularitide developed critical blood pressure reductions and overall developed more adverse reactions compared with participants randomized to placebo.

As patients with cirrhosis and portal hypertension develop arterial hypotension, they are sensitive to even slight unsteadiness of their neuro-hormonal equilibrium. This may explain the early decline in systolic blood pressure on initiation of ularitide infusion that plateaus after approximately 6 hours of exposure, paralleling observations in patients with decompensated heart failure.^16^ Furthermore, the kidney perfusion deteriorates early, most likely as a result of the systemic vasodilation induced by ularitide. A marginal increase in urine volume is observed initially for selected study subgroups (Supplemental Information http://links.lww.com/HC9/A946 and Supplemental Figure S4, http://links.lww.com/HC9/A946) coinciding with a modest reduction in the P-aldosterone concentration. However, this early response is small and not meaningful from a clinical perspective. Beyond 2 hours of treatment, the systolic blood pressure has declined to an extent critical for renal perfusion, expressed by increased P-creatinine.

Although this trial overall reached a negative conclusion, the results are important for patients with refractory cirrhotic ascites and managing physicians alike, as it contributes to new perspectives regarding the pathophysiology of cirrhotic ascites and the designing of future trials. The plasma concentrations of natriuretic peptides are elevated in patients with cirrhosis and extracellular fluid accumulation.^17,18^ However, when patients reach the far end of the spectrum of cirrhotic ascites, ie, refractory ascites, it may be suggested that the neuro-hormonal imbalance with severe activation of the renin-angiotensin-aldosterone-system and the sympathetic nervous system become too prominent for natriuretic peptides to reverse the pathological renal water and sodium retention.^13^ Our results support the concept of the renin-angiotensin-aldosterone-system activation constituting both a marker and critical mediator of renal resistance to vasodilators, as the P-aldosterone and P-copeptin concentrations increased after 24 hours of ularitide infusion. In addition, we observed the expected changes in P-cyclic GMP levels during ularitide treatment, demonstrating the pharmacokinetic effect of ularitide also in this patient group.^12^

In the trial planning phase, we estimated that 38 participants could be enrolled during 2 years. However, we experienced a slow recruitment rate mainly due to an unforeseen high percentage of exclusions due to preserved U-sodium excretion. Such patients may improve their ascites burden through better adherence to dietary salt restriction and compliance with medical therapies and may not have true “refractory” ascites. This appends the arguments for replacing the traditional binary ascites classification into refractory and diuretic-responsive with an ascites model that provides a broader description of ascites severity, suitable for clinical trials alongside improving ascites management in a clinical setting.^19^

Even though the trial was terminated early, the study design enabled comparison with a placebo group, along with preferring the collection of safety information on ularitide due to the 2:1 randomization allocation. Moreover, the trial design allowed dose titrations providing specific efficacy and safety data for ularitide doses higher (45 ng/kg/min) and lower (10 and 15 ng/kg/min) than the starting doses of 20 and 30 ng/kg/min; however, without indicating any dose window of optimal ularitide dosing to treat refractory ascites.

The study is limited by a short (2–4 hours) preinfusion period for baseline urine collection. As ularitide or placebo was administered as an add-on to diuretics, this relatively short baseline period can be influenced by the effects of diuretics, explaining the high baseline urine volume for 2 participants in the ularitide 30 ng/kg/min arm and one participant in the placebo arm. However, we chose not to stop spironolactone and furosemide before randomization to create a more clinically identical setting. Following clinical practice guidelines,^3,20^ patients with refractory ascites with a reasonable level of preserved renal sodium excretion rate are eligible for continued diuretic treatment at the highest tolerated dose alongside a moderate dietary sodium restriction. The latter recommendation extends the current dilemma of whether or not to pause diuretics before randomization with the choice of whether or not to streamline the dietary sodium intake before randomization. We chose not to change the patient’s diuretic treatment or dietary habits, both events that could disturb their neuro-hormonal equilibrium just before the initiation of the study treatment. Hence, this allowance of heterogeneity between participants is, to some extent, corrected with the individualized dosing scheme depending on the efficacy and safety of ularitide treatment. Finally, the study is limited by its small size, although the risk of drawing a false negative conclusion is negligible, favoring placebo rather than ularitide.

In conclusion, we investigated the safety and efficacy of the natriuretic peptide ularitide at doses of 20 and 30 ng/kg/min as a treatment of refractory ascites in patients with cirrhosis. The trial was terminated after safety interim analysis as the participants randomized to ularitide developed significant and persisting reductions in blood pressure that offset their renal responsiveness to the drug. Therefore, continuous i.v. ularitide infusions with doses of 20 and 30 ng/kg/min are unable to increase the urine volume and U-sodium excretion rate after 24 hours of treatment in patients with refractory cirrhotic ascites.

## Supplementary Material

SUPPLEMENTARY MATERIAL
