# Supplementary figures and images for: Randomized clinical trial on safety of the natriuretic peptide ularitide as treatment of refractory cirrhotic ascites
Source: Hepatol Commun. 2024 Jun 27;8(7):e0481. doi: 10.1097/HC9.0000000000000481 (PMC11213594; doi:10.1097/HC9.0000000000000481)

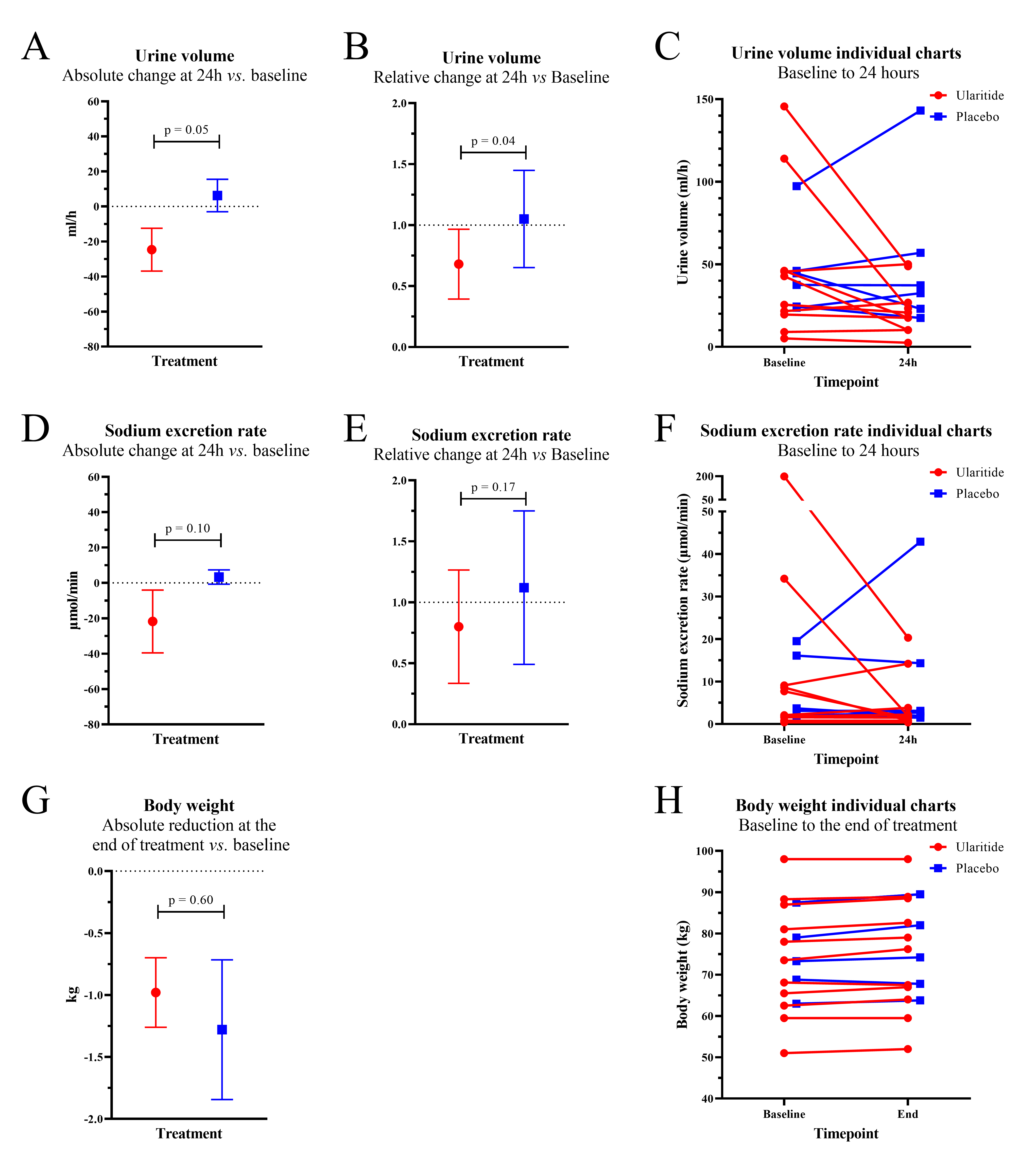

Supplement: SUPPLEMENTARY MATERIAL [file hc9-8-e0481-s001.tif]

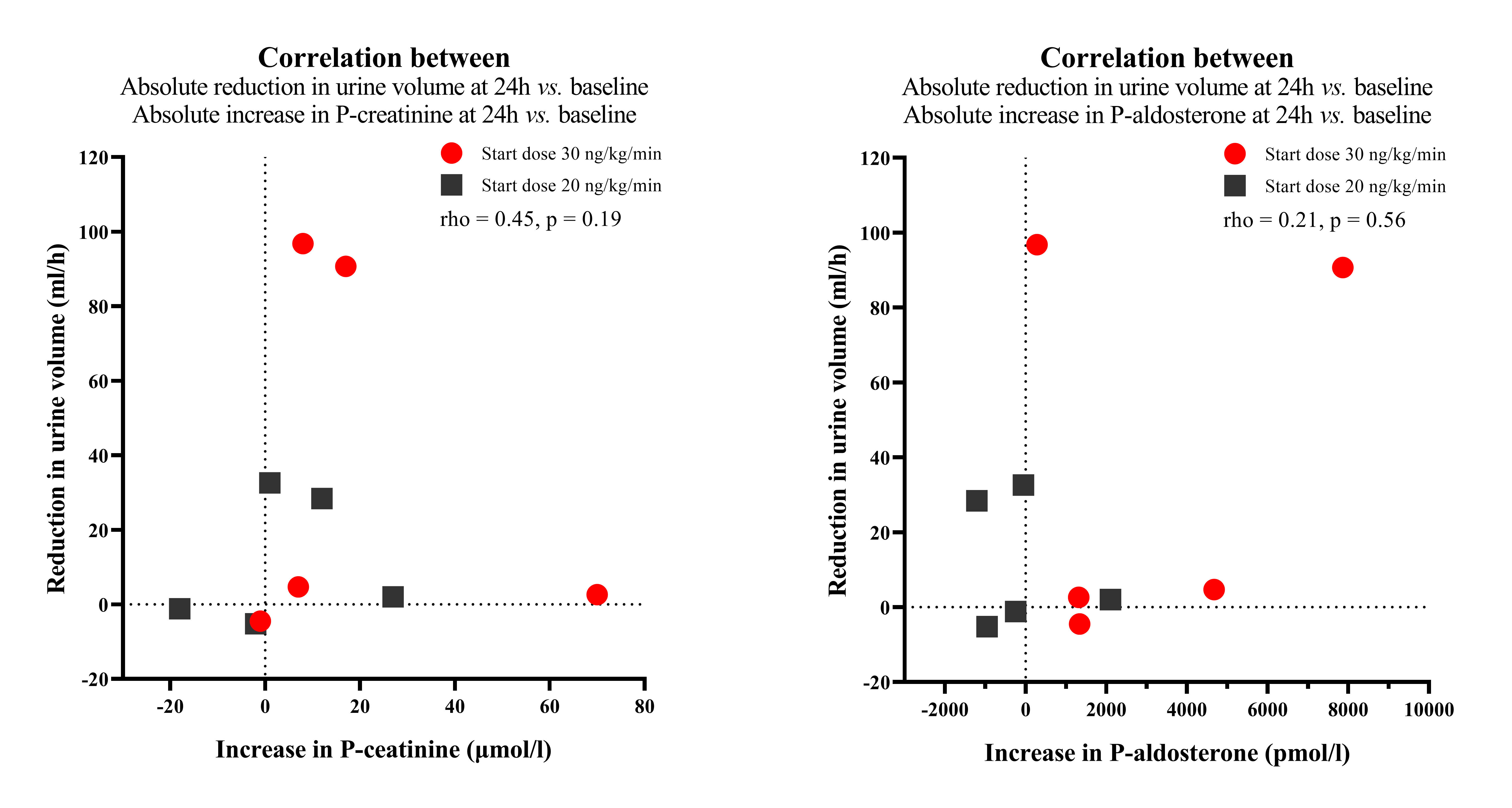

Supplement: SUPPLEMENTARY MATERIAL [file hc9-8-e0481-s002.tif]

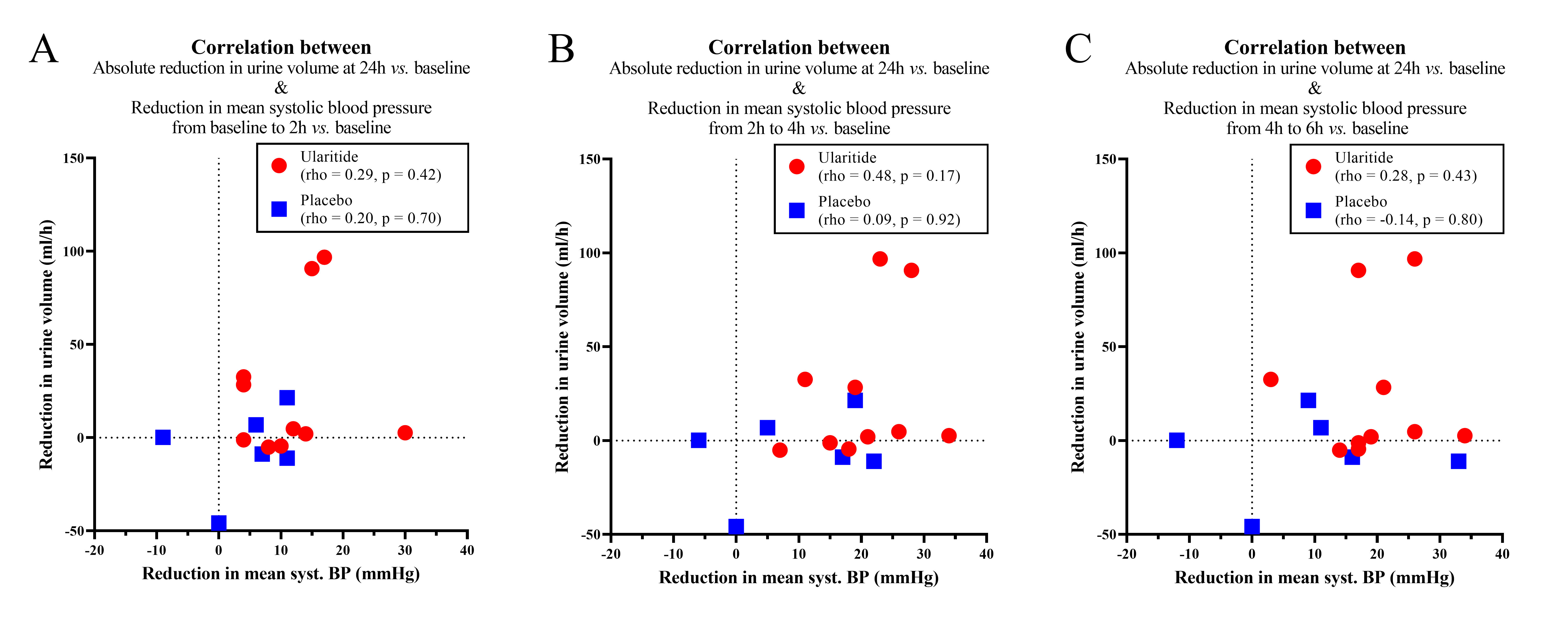

Supplement: SUPPLEMENTARY MATERIAL [file hc9-8-e0481-s003.tif]

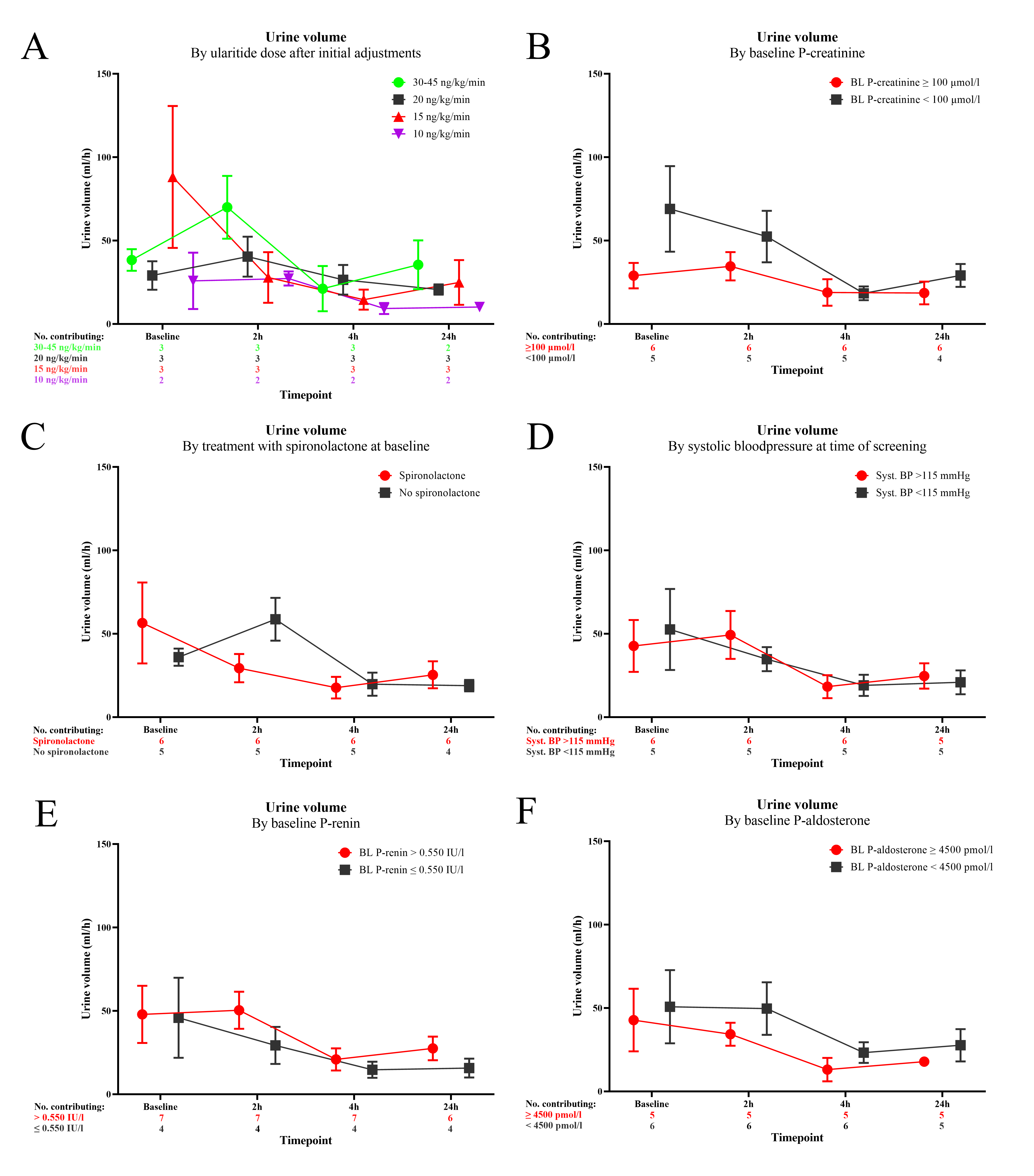

Supplement: SUPPLEMENTARY MATERIAL [file hc9-8-e0481-s004.tif]
